# Health outcomes, education, healthcare delivery and quality – 3038: The relation of sTRAIL levels and quality of life in omalizumab using severe persistent allergic asthma patients

**DOI:** 10.1186/1939-4551-6-S1-P212

**Published:** 2013-04-23

**Authors:** Arzu Didem Yalcin, Atil Bisgin

**Affiliations:** 1 Internal Medicine, Allergy and Immunology, Education and Research Hospital, Turkey; 2 Cancer institue., Sweden

## Background

Omalizumab represents a novel approach to the treatment of asthma, inhibiting the inflammatory cascade before it starts. As it was previously reported, the increase in the eosinophil levels in allergic asthma was due to the increase in peripheral blood eosinophil survival promoted by TRAIL. And as an apoptotic molecule TRAIL also present in cells that involved in asthma including eosinophils, mast cells, fibroblasts, and airway epithelial cells. It is expressed in airway remodeling and may be linked with the pathways of TGF-β1, which is thought to cause damage to the epithelium. The repair process of the epithelium is hindered as a result of increased apoptosis induced by TGF-beta1, which overlaps with the pathways of TRAIL. Moreover analogs of TRAIL could have therapeutical applications for asthma.

## Methods

In our previous report, we showed that sTRAIL levels of severe persistent allergic asthma patients were decreased after the anti-IgE treatment using omalizumab . Thus, the paper suggested that TRAIL may act as a soluble effector, and the decrease after the omalizumab treatment might be an indicator of clinical improvement. However, those results were had a limitation of follow-up period. Quality of life increased in the patients due to control of asthma with omalizumab treatment at the end of a year.

## Results

Moreover, we evaluated the sTRAIL levels again and compare them to the pre-treatment period and at the fourth month of the treatment. At the time of diagnosis, the mean sTRAIL level in the severe persistent allergic asthma patients before the omalizumab started was 1663 ± 120.4 pg/mL .At the fourth month, sTRAIL level decreased to 1443 ± 80.93 pg/mL . Finally, after a year of omalizumab usage, when all patients maintained significant improvement by a decrease in clinical symptoms and an increase in ACT, we found that sTRAIL levels were also decreased to very low levels 273±62.80 pg/mL .

## Conclusions

Our findings reflect the different mechanism(s) in the pathogenesis of allergic diseases by the regulation of both inflammatory system and apoptosis.

